# Post-Mortem Pedagogy: A Brief History of the Practice of Anatomical Dissection

**DOI:** 10.5041/RMMJ.10423

**Published:** 2021-01-19

**Authors:** Connor T. A. Brenna

**Affiliations:** Department of Medicine, University of Toronto, Toronto, ON, Canada

**Keywords:** Anatomy, dissection, epistemic frameworks, history, medical education

## Abstract

Anatomical dissection is almost ubiquitous in modern medical education, masking a complex history of its practice. Dissection with the express purpose of understanding human anatomy began more than two millennia ago with Herophilus, but was soon after disavowed in the third century BCE. Historical evidence suggests that this position was based on common beliefs that the body must remain whole after death in order to access the afterlife. Anatomical dissection did not resume for almost 1500 years, and in the interim anatomical knowledge was dominated by (often flawed) reports generated through the comparative dissection of animals. When a growing recognition of the utility of anatomical knowledge in clinical medicine ushered human dissection back into vogue, it recommenced in a limited setting almost exclusively allowing for dissection of the bodies of convicted criminals. Ultimately, the ethical problems that this fostered, as well as the increasing demand from medical education for greater volumes of human dissection, shaped new considerations of the body after death. Presently, body bequeathal programs are a popular way in which individuals offer their bodies to medical education after death, suggesting that the once widespread views of dissection as punishment have largely dissipated.

## INTRODUCTION

Anatomy, just like auscultation or analyzing a biopsy, is predicated on the idea that the body has a clinically insightful story to tell. Our willingness to listen to that story, however, bears a unique history grounded in the liminal and evolving thought-systems surrounding the body itself. Unlike other aspects of medical training, modern physicians study anatomy through a careful deconstruction of a human body, which is reliant on human dissection. Consequently, the practice of anatomy has seen a gradual transformation tied directly to the epistemic frameworks—dominated by religious and legal traditions—through which societies viewed the human body, and therefore also the ethics of dissection. In particular, a longstanding debate around the precise nature of the soul and the afterlife has been a catalyst for transforming policies encouraging and disavowing human dissection through history. Through the lens of how societies’ perceptions of the body shifted over time, modern practitioners can appreciate the lineage of anatomical dissection.

## THE FIRST ANATOMISTS

Herophilus, born in 335 BC, is recognized as the first person known to have performed and reported a systematic dissection of the human body.^1^,^2^ Along with his colleague Erasistratus, with whom he practiced in Alexandria (Egypt) under Ptolemaic permission, he was also accused centuries later by the Roman encyclopedist Celsus of performing vivisections on live subjects.^3^ Unfortunately, all of the written works of Herophilus are thought to have been lost in the fire that destroyed the fabled Library of Alexandria in 391 AD.^4^ The conditions at this particular time and place that promoted dissection as a useful educational tool relied on common ideas regarding the body’s significance after death. For example in Plato’s work *Phaedo*, which purportedly documents a conversation in 399 BC, the ancient philosopher Socrates suggests that when he dies his friends may do as they please with his body because his soul will leave it and live on eternally.^5^ His friends respond that many of their contemporaries in Ancient Greece believe that when an individual dies their soul no longer exists anywhere, but is destroyed.^5^ While these ideas were both compatible with human dissection, it was only permitted for less than half a century—Herophilus’s heyday—before new ways of thinking about the body post-mortem emerged.^6^ At this point, dissection is thought to have been entirely rejected by society.

## ANATOMY WITHOUT HUMAN DISSECTION

Many factors likely contributed to the reported outlawing of anatomical dissection in the third century BC.^7^ First, a new generation of empiricist physicians (of whom Filinos, ironically a disciple of Herophilus, was among the most prominent) popularized the belief that there was little practical utility in the study of anatomy.^8^,^9^ Second, a belief circulated in many cultures that interfering with the burial of a dead body was immoral, which may actually represent early public policy borne of a growing recognition that unburied bodies generated sickness.^10^,^11^ Finally, many ancient religions held beliefs that dissection would interfere with an individual’s salvation: it is commonly known, for example, that the ancient Egyptians believed that only if a corpse was properly embalmed and entombed could its former owner experience the afterlife.^12^ The rise of monotheistic religions led to the popularization of new ideological frameworks which held that the body’s condition of being “whole,” even after death, was of great religious consequence. Since such frameworks were incompatible with dissection, the practice was disavowed over the next 1500 years.^10^

During this period, the concept of anatomy continued to evolve without human dissection. Galen, a physician to gladiators, born in Pergamum (modern-day Turkey) in the year 129 AD, documented anatomical observations of his often seriously wounded charges to complement his extensive dissection of large animals such as monkeys and pigs.^13^ From these experiences he wrote anatomical treatises that served as the most influential source of anatomical knowledge in the world for well over a millennium.^13^,^14^ His dissections often had a theatrical public component, which boosted the popularity of his works.^15^ Galen operated within the belief that all structures were created for a purpose and that their study provided new ways to appreciate their creator, a belief which benefited from its compatibility with the major monotheistic (Jewish, Christian, and Muslim) religious views.^16^,^17^ Though Galen is still often considered the most accomplished researcher of antiquity, his treatises were anatomically imperfect, apparently relying too heavily on comparative animal dissections. For example, Galen described the human liver as having five lobes, when in reality human livers have only four—a pig’s liver has five lobes.^18^ Galen’s writings were translated into Arabic during the ninth century AD and spread through the Middle East, Europe, and India, where they would remain the dominant source of anatomical information for many centuries.^17^,^19^,^20^

## THE RENAISSANCE OF ANATOMICAL DISSECTION

The broad influence of Galen’s work contributed to another ideological development in the eleventh century AD—a recognition of the utility of anatomical knowledge in medicine, co-incident with the emergence of universities and the regulation of medical teaching, which was seen to outweigh at least some of the perceived ethical costs of dissection. In the year 1231, Frederick II (Emperor of the Holy Roman Empire from 1220 to 1250) decreed that at least one body could be dissected every five years for the education of physicians and surgeons.^21^ In 1315, Mondino de Luzzi, an Italian physician, went on to conduct what may have been the first public human dissection since Herophilus, on an executed criminal.^20^ This was not a unique occurrence: as dissection received legal support across Europe between the eleventh and fourteenth centuries, most countries had low limits on how many dissections could be performed per year and most allowed only criminals to be the subjects of dissection.^22^–^25^ Around the same time, the use of autopsy in criminal investigations was legitimized.^26^

This medieval anatomy was highly ritualized, with a professor reading Galen’s writings from a pedestal while assistants (often barber-surgeons) dissected a cadaver for an audience of students, and when differences were observed between a cadaver and the Galenic ideal they were attributed to the imperfections or sinfulness of the individual human subject rather than anatomical differences between humans and the animals Galen dissected.^13^ Dissection’s reservation for criminals may be an indication that society continued to view it as insalubrious until a second unique population of rare subjects emerged: those who died with an “odour of sanctity” (a sweet, floral scent associated with the death of a saint) and whose bodies were then dissected to look for physical signs of this sanctity.^27^

The narrow permissions surrounding human dissection during this era were available only for physicians, but the artist and polymath Leonardo da Vinci nevertheless became a preeminent anatomist at the beginning of the sixteenth century when he transitioned from the dissection of animal corpses to human bodies.^28^,^29^ During this time he produced hundreds of anatomical drawings from approximately 30 human dissections: his work was largely unpublished, but its posthumous discovery identified several novel discoveries about anatomical pathology (among them, atherosclerosis, hepatic sclerosis, and the structure of the coronary arteries).^29^,^30^ His contributions to anatomical dissection were curtailed by a papal decree in 1513 which banned him from carrying out this practice, ostensibly because he was not a physician but perhaps also because some of his speculations on embryos disagreed with ideas of the Church.^28^,^29^

The study of anatomy continued to develop in a setting of limited dissection until, in 1543, the Flemish physician Andreas Vesalius authored the influential treatise *De Humani Corporis Fabrica*. This was the first profusely illustrated, printed anatomy textbook, combining medical science with Renaissance illustration to make a visually compelling argument for the study of anatomy.^31^,^32^ Vesalius’s collection, grounded in human dissection, would rectify some of Galen’s widely propagated errors, but the theatrical and ritualistic way that anatomy was taught and learned would continue until dissection was performed exclusively for medical students.^33^ The creation of *De Humani Corporis* also continued to support the narrative that anatomy was a clinically useful knowledge.

## THE RESURRECTIONISTS AND THE ANATOMY ACT

The medical profession recognized the utility of anatomical dissection before the general public, and supply (limited by laws defining how many dissections could be performed in a given city per year) of criminal cadavers soon fell far short of demand. This prompted a dark chapter in the history of medicine, initiated by the Resurrectionists: students or hired hands who stole corpses from graves under the cover of night on behalf of the medical schools.^34^ These thefts were so well-known that they produced a variety of creative reactions by the bereaved in attempts to protect their loved ones’ remains, such as mort-safes, iron coffins, and graveyard watch patrols.^35^ A market was created for murderers like William Burke and William Hare, who co-operated a Scottish hostel in which they suffocated (the origin of the term “burking”) 16 victims and sold their bodies to a reputable professor of medicine at the University of Edinburgh, Dr Robert Knox.^36^,^37^ Once caught and tried in 1828, Hare sold out Burke, who was then hanged and publicly dissected as punishment for his crimes; his skeleton, as well as a pocketbook bound with his tanned skin, remain on display to this day in Edinburgh. Ultimately, the United Kingdom was forced to address the problem of body-snatching through the Anatomy Act of 1832, an act of parliament which allowed for the legal dissection of unclaimed bodies of poor citizens from workhouses and charitable hospitals (thereby introducing another troubling chapter in the history of anatomy).^34^,^38^,^39^ With a weakened association between dissection and criminality, however, perceptions that opening the body after death was a punishment reserved for the sinful began to vanish. Anatomical dissection then flourished under several generations of early surgeons and anatomists: the brothers John and William Hunter, for example, created many thousands of anatomical preparations which greatly influenced medical practice.^40^

## DISSECTION IN MODERN SOCIETY

The epistemic frameworks through which societies perceive the human body continue to evolve. Today, the concept of individual consequences if one’s body does not remain whole after death has fallen largely out of vogue in many cultures, creating the conditions necessary for dissection to feature prominently in undergraduate medical education. Rather than a punishment, body bequeathal is a gift that the profession is fortunate to receive from generous donors for medical education—and an opportunity that each of us possesses, as individuals, to give back.
